# Coronaviruses and gastrointestinal symptoms: an old liaison for the new SARS-CoV-2 

**Published:** 2020

**Authors:** Giacomo Caio, Lisa Lungaro, Rosario Cultrera, Roberto De Giorgio, Umberto Volta

**Affiliations:** 1 *Department of Morphology, Surgery and Experimental Medicine, University of Ferrara, Ferrara Italy*; 2 *Mucosal Immunology and Biology Research Center, Massachusetts General Hospital – Harvard Medical School, Boston, MA, USA*; 3 *Department of Medical and Surgical Sciences, University of Bologna, Bologna, Italy *

**Keywords:** Angiotensin-converting enzyme 2, COVID-19, Diarrhea, Gastrointestinal Presentation, Nausea, SARS-CoV-2, Vomiting.

## Abstract

The coronavirus disease (Covid-19) has caused a pandemic with more than 600,000 deaths to date. It is caused by the severe acute respiratory syndrome coronavirus 2 (SARS-CoV-2), a member of the beta-coronavirus genus that also includes SARS and the Middle East Respiratory Syndrome Coronavirus (MERS). While the typical presentation is given by respiratory symptoms and fever, some patients also report gastrointestinal symptoms such as diarrhea, nausea, vomiting, and abdominal pain. Several studies have identified the SARS-CoV-2 RNA in stool specimens of infected patients, and its viral receptor angiotensin-converting enzyme 2 (ACE2) is highly expressed in enterocytes. In this short review, we report the frequency of gastrointestinal symptoms in infected patients and suggest possible implications for disease management, transmission, and infection control.

## Introduction

 The novel coronavirus disease (COVID-19) has been spreading rapidly across the world, affecting more than 178 countries and causing more than 1.000.000deaths to date. COVID-19 is caused by the severe acute respiratory syndrome coronavirus 2 (SARS-CoV-2), a member of the Beta-coronavirus genus that also includes SARS-CoV and the Middle East Respiratory Syndrome Coronavirus (MERS-CoV). COVID-19 patients typically present with various respiratory manifestations ranging from mild flu-like symptoms (e.g., rhinitis, cough, sore throat, fever, joint/muscle pain) to severe life-threatening interstitial pneumonia (1 ▶). In addition, some patients report gastrointestinal (GI) symptoms such as diarrhea, nausea, vomiting, and abdominal pain in combination with the above-cited respiratory symptoms or, more rarely, as a unique manifestation of disease (2 ▶). Several studies have identified the SARS-CoV-2 RNA in stool specimens of infected patients, and its viral receptor angiotensin converting enzyme 2 (ACE2) is known to be highly expressed throughout the length of the gut mucosa, from mouth to rectum, with a higher expression in the small bowel and colon (3 ▶). These findings suggest that SARS-CoV-2 can actively infect and replicate in the GI tract, bearing possible implications for disease management, transmission, and infection control.

In this article, we aim to provide a rapid review of the most relevant gastrointestinal aspects of COVID-19 and highlight the implications through which knowledge of the relationship between SARS-CoV-2 and the gut may pave the way to future treatment methods. 

**Definition**

**Coronavirus family and the digestive system**

**Table 1:** Prevalence of gastrointestinal symptoms in SARS patients

Study	Patients	Diarrhea	Nausea/Vomiting	Abdominal pain
Booth CM et al.^15 ▶^	144	34 (23.6%)	28 (19.4)	5 (5.0%)
Liu CL et al.^16 ▶^	53	35 (66.0%)	6 (11.3%)	5 (9.4%)
Leung CW et al.^17 ▶^	44	9 (20.5%)	13 (29.5%)	4 (9.1%)
Jang TN et al.^18 ▶^	29	4 (13.8%)	5 (17.2%)	N/A
Lee N et al.^19 ▶^	138	27 (19.6%)	27 (19.6%)	N/A
Choi KW et al.^20 ▶^	267	41 (15.4%)	19 (7.1%)	N/A
Cheng VC et al.^21 ▶^	142	69 (48.6%)	N/A	N/A
Leung WK et al.^22 ▶^	138	53 (38.4%)	N/A	N/A
Kwan AC et al.^23 ▶^	240	49 (20.4%)	N/A	N/A
Peiris JS et al.^24 ▶^	75	55 (73.3%)	N/A	N/A
Total	1270	376/1270 (29.6%)	98/550 (17.8%)	14/241 (5.8%)

N/A not available

**Table 2:** Prevalence of gastrointestinal symptoms in MERS patients

Study	Patients	Diarrhea	Nausea/Vomiting	Abdominal pain
Arabi YM et al.^38 ▶^	330	38 (11.5%)	58 (17.6%)	47 (14.2%)
Choi WS et al.^39 ▶^	186	36 (19.4%)	26 (14.0%)	15 (8.1%)
Nam HS et al.^40 ▶^	25	8 (32.0%)	8 (32.0%)	8 (32.0%)
Assiri A et al.^30 ▶^	47	12 (25.5%)	10 (21.2%)	8 (17.0%)
Kim KM et al.^41 ▶^	36	7 (19.4%)	5 (13.9%)	N/A
Sherbini N et al.^42 ▶^	29	8 (27.6%)	8 (27.6%)	N/A
Saad M et al.^43 ▶^	70	21 (30%)	21 (30%)	17 (24.3%)
Almekhlafi GA et al.^44 ▶^	31	6 (19.4%)	4 (12.9%)	9 (29.0%)
Al Ghamdi M et al.^45 ▶^	51	13 (25.5%)	12 (23.5%)	N/A
Assiri A et al.^46 ▶^	23	5 (21.7%)	4 (17.4%)	N/A
Total	828	154/828 (18.6%)	156/828 (18.8%)	104/689 (15.1%)

N/A, not available.

Orthocoronavirinae or Coronaviruses (CoVs) are a sub-family of the Coronaviridae family, a group of single-stranded enveloped RNA viruses. They were identified during the 1960s and are known to infect animals and human epithelial cells in both the respiratory system and the GI tract (4 ▶-6 ▶). It is well known that CoVs sub-family viral shedding occurs via airways and the digestive system. For this reason, the transmission may occur not only through airborne droplets and fomites but also through the oro-fecal route (7 ▶). The CoVs sub-family is further subdivided into four genera, alpha, beta, gamma, and delta, based on their genetics and capacity to infect primarily (but not exclusively) mammals (alpha and beta) and birds (gamma and delta) (8 ▶). Until the end of 2019, only six species of CoVs wherewere known to infect humans, i.e. i) Coronavirus (CoV)-229E; ii) CoV-NL229E (from alpha-CoV genus); iii) CoV-OC43; iv) CoV-HKU1; v) Severe Acute Respiratory Syndrome (SARS)-CoV, and vi) Middle East Respiratory Syndrome (MERS)-CoV (all from beta-CoV genus). Notably, CoV-229E, -NL229E, -OC43, and -HKU1, also labelled as “Human CoVs unrelated to Severe Acute Respiratory Syndrome” (non-SARS HCoVs), are commonly isolated from children with acute gastroenteritis. Their significance as pediatric GI pathogens appears minor, however, as most of the HCoV findings in stools were co-infections with known gastroenteritis viruses (e.g., rotavirus and norovirus), but no definitive conclusions have been drawn (9 ▶).

**SARS and GI**

In 2003, SARS-CoV spread to over 30 countries, causing a respiratory disease with a mortality rate of about 8% (10 ▶). Phylogenetic analyses showed that the SARS-CoV genome is a result of a recombination of six different CoVs, namely: the porcine epidemic diarrhea virus (PEDV), transmissible gastroenteritis virus (TGEV), bovine coronavirus (BCoV), human CoV-229E, murine hepatitis virus (MHV), and the avian infectious bronchitis virus (IBV) (11 ▶). From a genetic standpoint, it is quite clear that SARS-CoV has a tropism for enterocytes, and as a result, GI symptoms were commonly reported in the SARS outbreak. About 20% of SARS patients had diarrhea on disease presentation (10 ▶, 12 ▶), and up to 73% presented with diarrhea mainly during the first week of disease with a mean duration of 3.7 days (13 ▶, 14 ▶). Analyzing the published papers reporting GI symptoms in SARS (Table 1), diarrhea was present in 376 out of 1270 studied patients (29.6% of cases, ranging from 13.8% to 73%), nausea in 79/408 (19.4%, ranging from 11.3% to 29.5%), vomiting 78/675 (11.5%, ranging from 9.4% to 29.5%), and abdominal pain in 14/241 (5.8%, ranging from 5.0% to 9.4% (Table 1) (15 ▶-24 ▶).

**Table 3:** Prevalence of gastrointestinal symptoms in COVID-19 patients

Study	Patients	Diarrhea	Nausea/Vomiting	Abdominal pain
Wang D et al.^54 ▶^	138	14 (10.1%)	14 (10.1%)	3 (2.2%)
Zhang JJ et al.^55 ▶^	139	18 (12.9%)	24 (17.3%)	8 (5.8%)
Guan W et al.^56 ▶^	1099	42 (3.8%)	55 (5.0%)	N/A
Zhou F et al.^57 ▶^	191	9 (4.7%)	7 (3.7%)	N/A
Chen N et al.^58 ▶^	99	2 (2.0%)	1 (1%)	N/A
Pan L et al.^59 ▶^	204	35 (17.1%)	4 (2%)	2 (1%)
Shi H et al.^60 ▶^	81	3 (3.7%)	4 (4.9%)	N/A
Lu X et al.^61 ▶^	171	15 (8.8%)	11 (6.4%)	N/A
Xu XW et al.^62 ▶^	62	3 (4.8%)	N/A	N/A
Huang C et al.^63 ▶^	38	1 (2.6%)	N/A	N/A
Liu K et al.^64 ▶^	137	11 (8%)	N/A	N/A
Yang X et al.^65 ▶^	52	0 (0%)	2 (3.8%)	N/A
Xu Y et al.^66 ▶^	45	0 (0%)	N/A	N/A
Zhao W et al.^67 ▶^	77	1 (1.3%)	6 (7.8%)	N/A
Xu H et al.^68 ▶^	1324	28 (2.1%)	N/A	N/A
Huang R et al.^69 ▶^	202	13 (6.43%)	N/A	N/A
Qi D et al.^70 ▶^	267	10 (3.7%)	6 (2.2%)	N/A
Yang P et al.^71 ▶^	55	2 (3.6%)	N/A	N/A
Shi S et al.^72 ▶^	645	29 (4.5%)	N/A	N/A
Luo S et al.^73 ▶^	1141	68 (6%)	134 (11.7%)	45 (3.9%)
Xu X et al.^74 ▶^	90	5 (5.5%)	5 (5.5%)	N/A
Lu H et al.^75 ▶^	265	17 (6.4%)	6 (2.3%)	N/A
Wen Y et al.^76 ▶^	417	29 (6.9%)	N/A	N/A
Yan S et al.^77 ▶^	168	12 (7.1%)	9 (5.3%)	7 (4.2%)
Ma L et al.^78 ▶^	81	6 (7.4%)	N/A	N/A
Yao N et al.^79 ▶^	40	3 (7.5%)	3 (7.5%)	N/A
Liu S et al.^80 ▶^	620	53 (8.5%)	N/A	N/A
Chen X et al.^81 ▶^	291	25 (8.6%)	17 (5.8%)	1 (0.3%)
Shu L et al.^82 ▶^	545	49 (9%)	0 (0%)	N/A
Liu L et al.^83 ▶^	153	14 (9.1%)	3 (2%)	1 (0.6%)
Fu H et al.^84 ▶^	36	3 (8.3%)	N/A	N/A
Zhao Z et al.^85 ▶^	75	7 (9.3%)	N/A	1 (1.3%)
Liu Y et al.^86 ▶^	109	12 (11%)	N/A	N/A
Fan L et al.^87 ▶^	55	6 (10.9%)	4 (7.3%)	N/A
Zhang JJ et al.^88 ▶^	139	18 (13%)	N/A	N/A
Fu H et al.^89 ▶^	52	7 (13.4%)	1 (1.9%)	N/A
Han R et al.^90 ▶^	108	15 (13.9%)	N/A	N/A
Ai J et al.^91 ▶^	102	15 (14.7%)	9 (8.8%)	3 (2.9%)
Wang L et al.^92 ▶^	18	3 (16.7%)	N/A	N/A
Lin L et al.^93 ▶^	95	23 (24.2%)	17 (17.9%)	2 (2.1%)
Chen Q et al.^94 ▶^	9	2 (22.2%)	0 (0%)	0 (0%)
Xu S et al.^95 ▶^	355	130 (36.6%)	N/A	N/A
Korea Centers for Disease Control and Prevention^96 ▶^	28	2 (7.1%)	0 (0%)	1 (3.6%)
Tabata S et al.^97 ▶^	104	10 (9.6%)	N/A	N/A
Cholankeril G et al.^98 ▶^	116	12 (10.3%)	12 (10.3%)	10 (8.6%)
Australia National Incident Room Surveillance Team^99 ▶^	295	48 (16.3%)	34 (11.5%)	6 (2%)
Dreher M et al.^100 ▶^	50	8 (16%)	2 (4%)	N/A
Young BE et al.^101 ▶^	18	3 (16.7%)	N/A	N/A
Kluytmans M et al.^102 ▶^	86	16 (18.6%)	15 (17.4%)	5 (5.8%)
Nobel YR et al.^103 ▶^	278	56 (20.1%)	63 (22.7%)	N/A
Hajifathalian K et al.^104 ▶^	1059	234 (22.1%)	168 (15.9%)	72 (6.8%)
Wolfel R et al.^105 ▶^	9	2 (22.2%)	N/A	N/A
Gritti G et al.^106 ▶^	21	5 (23.8%)	N/A	N/A
Pung R et al.^107 ▶^	17	0 (0%)	1 (5.9%)	N/A
Chen T et al.^108 ▶^	274	77 (28.1%)	24 (8.8%)	19 (6.9%)
Wan Y et al.^109 ▶^	230	49 (21%)	N/A	N/A
Xiao F et al.^110 ▶^	73	26 (35.6%)	N/A	N/A
Total	12648	1306/12648 (10.32%)	661/7727 (8.55%)	186/4373 (4.25%)

N/A not available

SARS-CoV was subsequently found in the feces of patients, and in some cases, the RNA presence persisted even after thirty days from disease onset (25 ▶). The SARS-CoV pathophysiological mechanism leading to cell infection and following viral replication occurs by binding of the envelope-anchored spike viral protein to a host receptor leading to a fusion of the SARS-CoV with targeted cell membranes. Evidence indicates that a defined receptor-binding domain (RBD) of SARS-CoV spike protein specifically recognizes the ACE2 expressed in type 2 alveolar cells as well as in the gut and several other tissues and organs (e.g., kidneys, endocrine tissues, liver, etc.) (26 ▶, 27 ▶).

**MERS and GI**

In 2012, MERS-CoV was identified as a zoonotic virus causing human respiratory disease (28 ▶) with 2494 cases (mainly in Saudi Arabia and Korea) and 858 deaths (34.4% mortality rate) (29 ▶). GI symptoms were the most commonly reported extrapulmonary clinical features of MERS, and about one-third of patients suffered from abdominal pain, nausea, vomiting, and diarrhea (30 ▶, 31 ▶). GI symptoms were present at disease onset in about 25% of patients, some of whom experienced fever and GI complaints before respiratory symptoms (30 ▶, 32 ▶). MERS-CoV RNA was found in patients’ feces in about 15% of cases (33 ▶). The mechanism used by MERS-CoV to infect and replicate in human cells occurs via the dipeptidyl peptidase 4 receptor (DPP4), a protein mainly expressed by endothelium and enterocytes, and even in the blood in a soluble form (34 ▶). Experimental studies have demonstrated that MERS-CoV could replicate primarily in enterocytes via DPP4 and then spread to the lungs, which suggests that in some cases, pneumonia is secondary to intestinal infection (35 ▶). Human intestinal epithelial cells are highly susceptible to MERS-CoV, providing a strong viral replication environment. This would explain some cases of MERS caused by the consumption of unpasteurized camel milk or undercooked camel meat via the oro-fecal route (36 ▶). Indeed, MERS-CoV RNA could be detected in 41.7% of milk samples collected from lactating camels, which shed the virus in nasal secretion and/or feces (35 ▶, 37 ▶). The prevalence of GI symptoms in MERS patients is summarized in Table 2 (30 ▶, 38 ▶-46 ▶).

**COVID-19 and GI**

At the end of 2019, a new coronavirus was identified as the etiological agent of a cluster of interstitial pneumonia cases in the Chinese city of Wuhan (47 ▶). The World Health Organization and Coronaviridae Study Group of the International Committee on Taxonomy of Viruses termed the novel coronavirus SARS-CoV-2, and the related disease was referred to as “COronaVIrus Disease 2019” (hence, ‘COVID-19’) (47 ▶, 48 ▶). The genome sequence of SARS-CoV-2 is 98% similar to SARS-CoV-1 and 50% similar to MERS (49 ▶). Both SARS-CoV and SARS-CoV-2 encode and express the spike protein that binds to the ACE2 receptor to enter human cells (27 ▶, 50 ▶). The RBD domain confers higher affinity to SARS-CoV-2 than SARS-CoV to bind the human ACE2, a feature which correlates with the efficient spread of the virus among humans and the ability to infect tissues even with low ACE2 expression (51 ▶, 52 ▶).

Based on these pathophysiological mechanisms and the previously quoted molecular and clinical studies performed on SARS-CoV-1 and SARS-CoV-2, it is quite clear that the new virus can infect enteric cells and be spread via the oro-fecal route (10 ▶, 12 ▶-14 ▶, 25 ▶-27 ▶, 49 ▶-52 ▶). The tropism for the GI tract has been confirmed by staining visualization of the viral nucleocapsid proteins of SARS-CoV-2 in the cytoplasm of gastric, duodenal, and rectal epithelial cells (53 ▶). Although at a lower frequency than SARS and MERS, some COVID-19 patients show GI symptoms such as diarrhea, nausea/vomiting, and abdominal pain during the course of the disease, although rarely as a unique manifestation (2 ▶) (Table 3) (54 ▶-110 ▶).

Explanations for the different frequencies of GI clinical manifestations can be found in the complex pathogenesis, in the host-virus interaction mediated by the immune system, and the different microbiota composition in the gut and airways (111 ▶).

The ACE2 expression in the gut is not intrinsically a condition sufficient for the virus to enter into enterocytes. Several other proteins of the host are involved in the ACE2 signalling network, such as the transmembrane protease serine 2 (TMPRSS-2), cathepsin B (CTPB), cathepsin L (CTPL), DPP4, aminopeptidases (ANPEP), monocyte chemoattractant protein-1 (MCP-1/CCL2), transferrin receptor (TFRC), meprin A subunit alpha (MEP1A), disintegrin, metalloproteinase domain 17 (ADAM17), fatty-acid binding protein 2 (FABP2), intracellular cholesterol transporter (NPC1), and C-Type Lectin Domain Family 4 Member M (CLEC4M) (112 ▶). Recently, it was demonstrated that the virus could enter human cells without using ACE2. An alternative receptor, CD147, has been described as a new route for SARS-CoV-2 infection, thus bypassing ACE2 and its pathway (113 ▶). Taken this information together, it is quite clear that SARS-CoV-2 infection and damage to GI cells occur through a variety of molecular targets rather than being mediated solely via ACE2. Indeed, following the latter erroneous hypothesis, the gut would be the main target for COVID-19, as it expresses 70 times more ACE2 than the lungs (3 ▶), whereas intestinal symptoms are present in less than 15% of COVID-19 patients.

The origin of GI symptoms in patients affected by COVID-19 remains to be clarified, as many patients with the live virus present in stools do not have intestinal complaints (114 ▶). Despite the presence of live virus in the stools, the oro-fecal spreading mechanism has not been fully demonstrated. Most patients may have very little traces of non-vital virus RNA in feces detected by RT-PCR, and only a minority have a vital virus at a low viral load unable to infect a new host (2 ▶). For this reason, there is still inadequate evidence to support stool testing for the diagnosis or monitoring of COVID-19 patients. Even the GI symptoms are of uncertain origin in patients with COVID-19, being caused by active viral replication in the gut in some patients or attributed to other causes, including pharmacologic treatments such as non-steroidal anti-inflammatory drugs (NSAIDs), antibiotics (azithromycin), and antiviral agents. In this context, the likely occurrence of gut dysbiosis, i.e. changes in the richness and diversity of the normal composition of the gut microbiota, along with altered epithelial cell permeability may participate to GI symptom generation in patients with COVID-19. One may think that GI symptoms result from an impaired anatomo-microbiological functional barrier constituted by enterocytes, tight-junctions, mucus, microbiota, and the immune system triggered by SARS-CoV-2 infection acting in concert with other noxious factors, e.g., other pathogens and/or drugs (some of them commonly used in clinical practice, such as NSAIDs) (115 ▶). The alteration of the intestinal barrier and dysbiosis along with gastrointestinal infections and psychosocial distress are the main pathophysiological mechanisms underlying functional GI disorders (FGIDs) such as post-infectious irritable bowel syndrome (PI-IBS) and functional dyspepsia (PI-FD) (116 ▶). COVID-19 is a paradigm for the proposed pathogenetic mechanisms, involving post-infective gut dysbiosis along with psychosocial distress due to the lockdown and loss of relatives and work. Clearly, further studies are necessary to establish the impact of the COVID-19 pandemic on the new onset of IBS and FD. 

## Conclusions

In conclusion, physicians should avoid overlooking or under evaluating GI symptoms in COVID-19 patients. The primary aim is to manage nausea, vomiting, and diarrhea via symptomatic treatment options along with the use of probiotics to limit or control the occurrence of intestinal dysbiosis. In daily practice, it is recommended to exclude other common causes of intestinal symptoms, such as *Clostridium difficile* infection, particularly in hospitalized patients. Finally, since GI symptoms alone are quite rare in COVID-19 patients (being detectable in less than 3% of cases), routine SARS-CoV-2 stool test is not indicated and, to the best of our knowledge, it should be performed only in patients with negative naso-pharyngeal swabs in the presence of clear imaging features indicative of interstitial pneumonia.
